# Racial disparities in calculated risk for bronchopulmonary dysplasia: a dataset

**DOI:** 10.1016/j.dib.2020.105674

**Published:** 2020-05-11

**Authors:** Zachary A. Vesoulis, Christopher C. McPherson, Halana V. Whitehead

**Affiliations:** Department of Pediatrics, Division of Newborn Medicine, Washington University School of Medicine

**Keywords:** Bronchopulmonary dysplasia, Prematurity, Racial disparity, Steroids

## Abstract

Bronchopulmonary dysplasia (BPD) is a severe pulmonary complication of prematurity and is associated with significant morbidity or death. Early use of systemic corticosteroids may alter the trajectory of the disease and improve outcomes. A BPD Outcomes estimator, developed by the NICHD using a large population dataset, can be used to calculate individual risk. Risk above a certain threshold may indicate that the benefits of corticosteroids outweigh the risks. Empiric analysis of this calculator by systematic entry of synthetic patient information reveals a marked racial disparity; black infants have lower risk of moderate/severe BPD due to a higher risk of death despite equivalent severity of illness. Interpretation and analysis of this finding can be found in “The challenge of risk stratification of preterm infants in the setting of competing and disparate healthcare outcomes” [1].

In this report, we provide the underlying data used in this analysis. Calculator output for 108 example patients, systematically varied by sex, birthweight, race, type of ventilator, and fraction of inspired oxygen (FiO_2_), is reported.

Specifications Table

**Table:** 

Subject	Perinatology, Pediatrics and Child Health
Specific subject area	BPD risk prediction in preterm infants
Type of data	Table
How data were acquired	Output from the NICHD BPD Outcomes calculator (https://neonatal.rti.org/index.cfm?fuseaction=BPDCalculator.start)
Data format	Raw data; XLS format
Parameters for data collection	108 synthetic patients, systematically varied by birth weight, sex, race, ventilator mode, and FiO_2_. Gestational age was held constant at 24 weeks.
Description of data collection	Column 1 (id_number): unique identifier Column 2 (gestational_age): gestational age in completed weeks Column 3 (birthweight): Birth weight in grams Column 4 (sex): infant sex Column 5 (race): infant race, categorized as white, black, or Hispanic Column 6 (day_of_life): age of infant in days Column 7 (vent): method of respiratory support, listed as synchronized intermittent mandatory ventilation (SIMV) or HFOV (high-frequency oscillatory ventilation) Column 8 (fio2): fraction of inspired oxygen (percentage) Column 9 (death_risk): calculated risk of death (percentage) Column 10 (severe_bpd_risk): calculated risk of having severe BPD (percentage) Column 11 (mod_bpd_risk): calculated risk of having moderate BPD (percentage) Column 12 (mild_bpd risk): calculated risk of having mild BPD (percentage) Column 13 (no_bpd_risk): calculated risk of having no BPD (percentage) Column 13 (mod_severe): combined risk of having moderate or severe BPD
Data source location	Institution: Washington University City/Town/Region: St. Louis, MO Country: USA
Data accessibility	Repository name: Mendeley Data Data identification number: [provide number] Direct URL to data: http://dx.doi.org/10.17632/v58vmxznk3.1[2]
Related research article	Whitehead HV, McPherson CC, Cohlan BA, Rao R, Vesoulis ZA, Warner BB, Cole FS. The challenge of risk stratification of preterm infants in the setting of competing and disparate healthcare outcomes. The Journal of Pediatrics. *Accepted for publication. [**1**]*

## Value of the data

• These data highlight an obstacle in application of the output of the NICHD BPD Outcomes estimator because black infants have a consistently lower predicted risk of BPD due to a higher risk of mortality.
• Neonatologists, Pulmonologists, and Pharmacists developing postnatal corticosteroid treatment algorithms will benefit from the knowledge that this disparity exists.
• These data suggest that applications of BPD risk calculators should incorporate the difference in mortality risk by use of an alternate threshold or an expanded metric.
• These data further demonstrate that sociodemographic factors can have a powerful influence on NICU outcomes, and that care should be taken when developing calculators or guidelines to ensure that disparities which drive differences in outcomes are accounted for in risk assessment.

## 1 Data Description

A single table of data is provided. Each row represents a single patient, with systematic variation of each clinical factor except for gestational age (which is held constant at 24 weeks). Column 1 is a unique identifier, columns 2-8 encompass clinical and demographic factors, while columns 9-14 provide the calculated risk of death, severe BPD, moderate BPD, mild BPD, no BPD, and combined risk of moderate/severe BPD.

## 2 Experimental Design, Materials, and Methods

These values were obtained from the NICHD BPD Outcomes estimator (https://neonatal.rti.org/index.cfm?fuseaction=BPDCalculator.start). This tool uses clinical and outcome data collected between 2000-2004 from 3629 preterm infants born before 30 weeks gestation [3]. The web-based tool accepts the following data points: gestational age, birth weight, sex, race [white, black, Hispanic], postnatal age, ventilator type, and FiO_2_. Based on these data, the calculator then outputs the predicted probability of five different outcomes (death, severe BPD, moderate BPD, mild BPD, no BPD).

The goal of this project was to systematically vary the input variables to evaluate the calculator response. Two variables were held constant in all trials (gestational age at 24 weeks, day of life at 7 days), while sex, race, ventilator type, and FiO_2_ were serially incremented. Possible values for sex were male and female; for race were white, black, and Hispanic; for ventilator type were synchronized intermittent mandatory ventilation (SIMV) and high-frequency oscillatory ventilation (HFOV); FiO_2_ was incremented in 10% blocks from 21 to 100%.

The calculated risk of each of the 5 outcomes was recorded in the table. A meta-analysis by Doyle [4] suggests that systemic postnatal corticosteroids should be considered when the combined risk of moderate and severe BPD exceeds 60%. For that reason, the sum of these two risks was added as an additional column.

## Appendix Supplementary materials

Image, application 1
